# Pathogenic type-2 immunity beyond allergy: the role of Th2 cell heterogeneity in helminth infections

**DOI:** 10.1128/iai.00556-25

**Published:** 2026-07-13

**Authors:** Camila Queiroz-Glauss, Pedro H. Gazzinelli-Guimaraes

**Affiliations:** 1 Laboratory of Translational Type 2 Immunity, Department of Microbiology, Immunology and Tropical Medicine, George Washington School of Medicine and Health Sciences 43989, Washington, DC, USA; University of California Merced, Merced, California, USA

**Keywords:** helminth, allergy, type 2 immunity, Th2 cells, immune regulation

## Abstract

Type 2 immunity is a defining feature of helminth infection and is traditionally viewed as protective, promoting parasite expulsion, tissue repair, and/or suppression of bystander antigens. However, work in allergic disease has demonstrated that Th2 cells are not functionally uniform but instead comprise specialized subsets, including highly differentiated pathogenic Th2 populations characterized by sustained IL-5/13 production, epithelial alarmin responsiveness, and stable tissue residency. The presence of these cells is associated with immunopathology and worse clinical outcomes. Interestingly, many helminth-associated pathologies, including granulomatous fibrosis in schistosomiasis, pulmonary injury during *Ascaris* larval migration, and eosinophil-driven inflammation in filariasis, mirror mechanisms attributed to pathogenic Th2 cells in allergy. Yet Th2 responses in helminth infections remain largely defined by bulk cytokine production rather than discrete cellular programs. Here, we synthesize emerging evidence from human infection studies and experimental models demonstrating the expansion of highly differentiated Th2 subsets during helminth infection that phenotypically and transcriptionally resemble pathogenic Th2 cells defined in allergy. We examine how these subsets associate with tissue pathology, symptom severity, or altered immune responsiveness, while also highlighting important context-dependent differences in regulatory and effector balance. This review challenges the traditional view of helminth-induced Th2 immunity as a uniform response and instead supports a model in which specialized Th2 programs differentially contribute to protection, pathology, and immune modulation during infection. We propose that resolving this heterogeneity through high-dimensional and spatial approaches will be essential to redefine helminth-induced type 2 immunity and to distinguish protective from pathology-driving programs within host-parasite interactions.

## INTRODUCTION

Historically, type 2 immunity was conceptualized primarily as a host defense response directed against large multicellular parasites which cannot be killed through classical phagocytic mechanisms. Within this framework, Th2 responses were largely viewed as promoters of humoral immunity, with IL-4-driven B cell maturation and class switching to IgE and IgG4 considered their defining functional outputs (1, 2). Over the past two decades, however, this simplified model has been substantially revised. A growing body of evidence from both allergic disease and helminth infection has demonstrated that type 2 polarized CD4^+^ T cells orchestrate a far broader range of effector mechanisms, directly regulating diverse target populations including mast cells, basophils, eosinophils, macrophages, and epithelial cells (3). This expanded understanding has reframed Th2 immunity as a multifaceted tissue-directed program rather than a predominantly antibody-focused response (4). While this expanded type 2 immune program involves multiple cellular sources, including type 2 innate lymphoid cells (ILC2s), CD4^+^ Th2 cells, eosinophils, M2 macrophages, epithelial cells, mucus production, mast cells, basophils, and IgE, the present review focuses specifically on CD4^+^ T cell heterogeneity in allergic diseases and helminth infections.

Although both allergic diseases and helminth infections are characterized by type 2-biased immunity, the degree to which Th2 cells’ heterogeneity has been operationally defined differs markedly between these contexts. In allergic disease, multiple studies have established the existence of highly differentiated, pathogenic Th2 populations that are phenotypically, transcriptionally, and functionally distinct from conventional Th2 cells and that are closely linked to disease severity and chronicity (5–10). These pathogenic Th2 cells—also referred to as pTh2, peTh2, Tpath2, or proallergic (Th2A)—are recognized as a discrete Th2 subset with enhanced effector function (10), with differences in nomenclature largely reflecting experimental context and discovery platform rather than fundamental biological divergence. Across studies, their identification converges on a core set of surface and signaling markers, including CCR8 (11), IL17RB (9, 12, 13), IL9R (12, 13), KLRB1 (CD161) (9, 12, 14), Prostaglandin D₂ Receptor 2 (PTGDR2, commonly known as CRTH2) (9, 12), together with a broadened cytokine profile characterized by high IL-13 (9, 12) and IL-9 (9) production and, most prominently, sustained IL-5 expression (9, 12, 14). These pathogenic Th2 programs are preferentially expanded in atopic individuals (9), correlate with eosinophilic inflammation (15, 16) and tissue pathology (11–13, 17), and are frequently maintained by epithelial-derived signals, supporting their role in chronic and severe allergic disease.

In contrast, although helminth infections robustly induce type 2 cytokine environments, most helminth studies, particularly in humans, continue to describe Th2 responses as a relatively homogeneous compartment defined primarily by bulk IL-4, IL-5, and IL-13 production (18). However, the functional consequences of Th2 immunity in helminth infection are highly context-dependent and extend far beyond a uniform effector program. Depending on the parasite species, tissue niche, and stage of infection, type 2 responses can mediate protection, contribute to immunopathology, promote tissue repair, or regulate co-infection and/or bystander inflammatory responses (3, 19–26). In several models, Th2 cytokines are essential for parasite control, as IL-4- and IL-13-driven mechanisms facilitate expulsion of gastrointestinal nematodes and correlate with reduced worm burden in human hookworm infection (19, 20). In contrast, exaggerated or dysregulated Th2 responses can also drive disease, exemplified by IL-13-dependent granuloma formation and fibrosis in schistosomiasis, and by eosinophil and mast cell-mediated pathology in filarial infections (21, 22, 27). At the same time, type 2 immunity plays a critical reparative role, promoting alternatively activated macrophage responses and epithelial regeneration that restore tissue integrity following parasite-induced damage (3). Moreover, during chronic helminth infections, and other gastrointestinal parasitoses, such as giardiasis, Th2 cells become a major source of IL-10, alongside Foxp3^+^ regulatory T cells, and play a critical role in downregulating immune responses not only against the persistent parasite itself but also against bystander antigens and inflammatory conditions, including viral, protozoa, and bacterial infections as well as autoimmune diseases (26, 28–32). However, the role of IL-10 in helminth infection is not exclusively immunosuppressive; recent work has demonstrated that IL-10 signaling can also promote Th2 differentiation and enhance anti-parasite immunity by suppressing competing Th1 responses in infected intestinal tissue (24). In addition, IL-10-producing Th2 cells are required for effective worm expulsion during primary infection, by promoting intestinal goblet cell differentiation and increased generation of M2 macrophages (33).

Despite this functional complexity, the cellular heterogeneity underlying helminth-induced Th2 responses remains poorly defined. As a result, it remains unclear whether pathogenic Th2 programs analogous to those described in allergy are absent, actively suppressed, or simply under-characterized during acute and/or chronic helminth infection (6). This discrepancy underscores an important conceptual and technical gap in the field and suggests that applying allergy-derived frameworks of Th2 heterogeneity to helminth infections may yield critical insights into how type 2 immunity is differentially programmed toward pathology vs immune regulation.

## PATHOGENIC Th2 CELLS

### Definition

The concept of pathogenic Th2 cells emerged from early observations, in human and mouse studies, that not all Th2 differentiated cells were phenotypically homogeneous, particularly regarding IL-5 expression (6, 34). These studies identified at least two distinct Th2 subsets—one capable of producing IL-5 and another lacking IL-5 expression (15, 34). Alongside the knowledge that IL-5 is critical to eosinophil development, activation, and survival (35), this heterogeneity suggested that not all Th2 cells would contribute equally to disease pathogenesis, since they can be driven through eosinophil-dependent (36), IL-4/IL-13 signaling axis (37), or IgE-mediated pathways (38). Indeed, human IL-5 producing Th2 responses were preferentially associated with eosinophil-dominant conditions, such as eosinophilic gastroenteritis, whereas IL-5 negative Th2 responses were more commonly observed in IgE-mediated disorders, including food allergy (15).

Multiple studies subsequently linked the selective expansion of IL-5-producing Th2 subsets with increased tissue pathology and inflammation in allergic settings, including mouse models of atopic dermatitis (11) and asthma (39). However, it was not until 2014 that the term pathogenic Th2 cells was formally introduced to describe a distinct Th2 subpopulation with unique functional properties (7).

This concept is now widely used to define a specialized subset of CD4^+^ Th2 cells characterized by stable effector differentiation and a disproportionate capacity to drive tissue inflammation and immunopathology (17, 40, 41). The presence of these cells is clinically associated with increased disease severity, chronicity, and resistance to standard therapies (6, 9, 13, 16, 42–44). It is this tight linkage between stable effector function and pathogenic outcome that distinguishes pathogenic cells from conventional Th2 populations, which produce type 2 cytokines without inducing overt tissue damage (45). These cells have been variably termed pTh2, peTh2, Tpath2, or Th2A in the literature and, despite differences in proposed surface markers or experimental context, are generally considered to represent overlapping manifestations of the same pathogenic Th2 compartment.

### Identification

The identification of core markers that reliably define pathogenic Th2 cells not only is essential for dissecting the biology and clinical relevance of this population across disease contexts but also represents an innovative and transformative strategy to identify potential therapeutic targets, addressing the unmet need for treatments capable of reversing chronic tissue damage and restoring function.

However, much like the nomenclature itself, the surface and signaling markers used to identify pathogenic Th2 cells are not applied uniformly across the literature. Instead, different studies emphasize partially overlapping phenotypic, transcriptional, and functional features, often shaped by experimental system, tissue compartment, disease context, and species (mouse or human). As a result, no single marker—or fixed combination of markers—has emerged as universally definitive. Rather, best practice has evolved toward defining pathogenic Th2 cells through the integration of a conserved set of core markers with contextual cues, including cytokine production profiles, differentiation state, tissue localization, and disease-specific associations.

One of the earliest and most consistent features attributed to pathogenic Th2 cells was their classification as a highly differentiated effector population. This was initially inferred, in mice, from reduced expression of the lymphoid homing receptor L-selectin (CD62L), together with low expression of the chemokine receptor CXCR3 (39), indicating diminished recirculation through secondary lymphoid tissues (46). In parallel, mouse transcriptional profiling revealed selective downregulation of the T-box transcription factor Eomesodermin (Eomes) in IL-5 producing Th2 cells, despite its higher expression in other memory Th2 subsets, linking stable IL-5 production to a terminally differentiated effector state (39). In human studies, the absence of the expression of markers associated with naïve or early memory states, such as CD27 (47–52) and CD45RA (and, depending on the framework, CD45RB) (43, 49, 50), is usually used as a first step to identify pathogenic Th2 cells.

Subsequent studies across both murine models and human Th2-mediated diseases expanded this phenotypic framework by identifying surface markers associated with pathogenicity, tissue residency, and survival. In mouse atopic dermatitis, the chemokine receptor CCR8 emerged as a defining marker, following observation that high expression of its ligand, CCL8, in inflamed skin selectively promotes the recruitment and accumulation of CCR8^+^ IL-5 producing Th2 cells at sites of disease (11).

Broader analyses with human and mouse samples encompassing chronic rhinosinusitis, eosinophilic gastrointestinal disease, and environmental allergies further characterized pathogenic Th2 cells by their expression of epithelial alarmin receptors, including IL-17RB (IL-25R), ST2 (IL-33R), and TSLPR (12, 53–56). ST2 expression is associated with a transient phenotype, as it enables Th2 cells to respond robustly to elevated levels of IL-33, which are abundant in allergic tissues. Because IL-33 signaling is known to amplify Th2 effector functions (57), ST2 expression further enhances the pathogenic potential of these cells (58).

In humans, the prostaglandin D₂ receptor CRTH2 and the C-type lectin receptor CD161 are among the most commonly used markers to identify pathogenic Th2 cells across disease contexts (12, 43, 47, 48, 55, 59–61). CRTH2 expression is linked to enhanced Th2 cell migration and activation, as prostaglandin D₂–CRTH2 signaling promotes upregulation of adhesion molecules, exacerbated secretion of type 2 cytokines (62, 63), and has been correlated with increased IgE levels (60). In addition to surface CRTH2 expression, several studies have identified high intracellular expression of hematopoietic prostaglandin D₂ synthase (HPGDS) as a complementary and functionally informative marker of pathogenic Th2 cells (5, 64, 65). HPGDS catalyzes the terminal step in prostaglandin D₂ biosynthesis, indicating that these cells are not only responsive to prostaglandin D₂ signaling but also represent an intrinsic cellular source of this lipid mediator (59). On the other hand, CD161 is strongly associated with memory T cell populations (66). Through its interaction with its ligand Lectin-like transcript 1 (LLT1), CD161 mediates IL-2 secretion which reduces susceptibility to apoptosis, thereby supporting prolonged survival of pathogenic Th2 cells (5).

**Fig 1:**
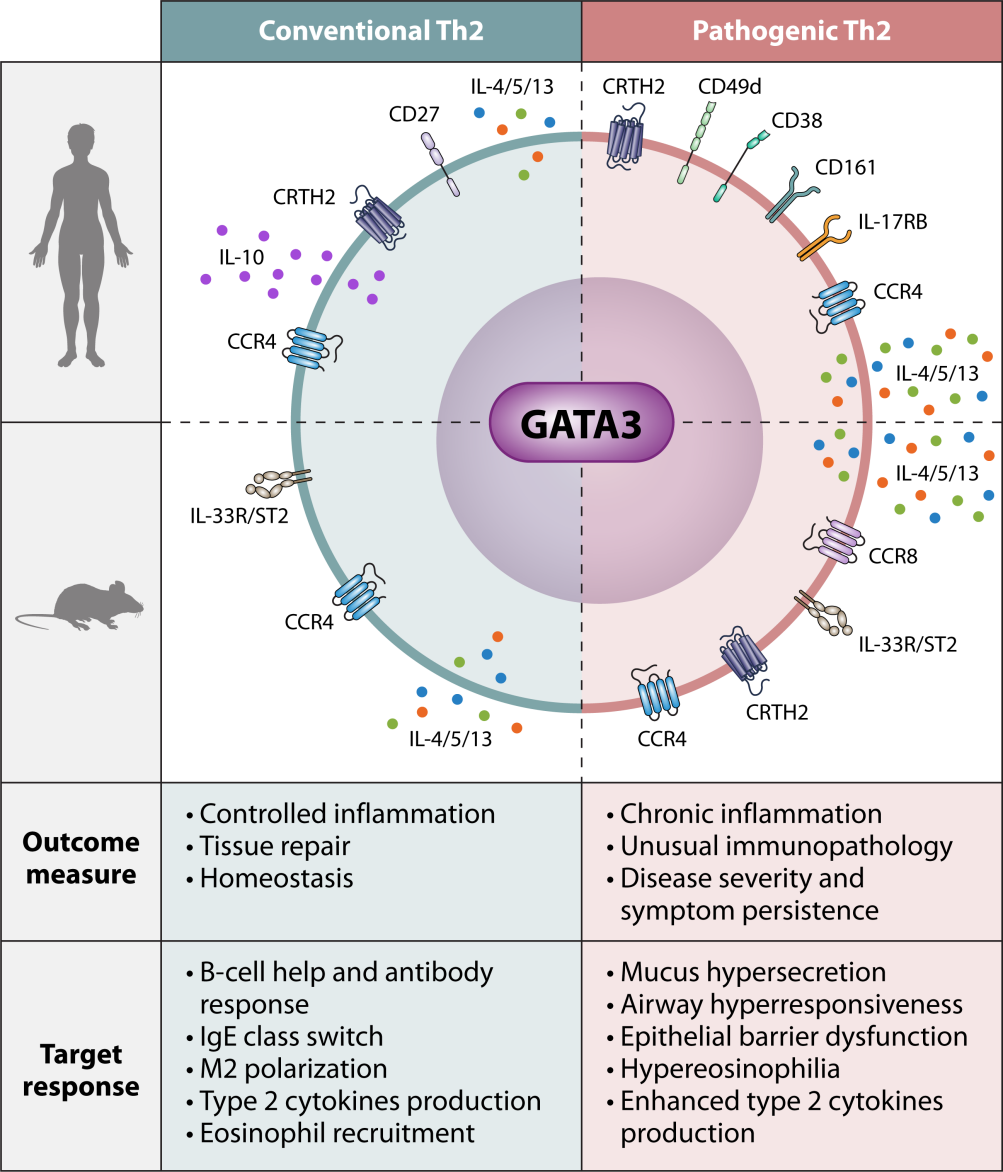
Phenotypic markers distinguishing conventional and pathogenic Th2 subsets in humans and mice. Schematic representation of canonical intracellular and surface markers of conventional (left) and pathogenic (right) Th2 cells in humans (top) and mice (bottom).

Complementary markers such as CD49d, associated with tissue retention and allergen specificity (9), IL-9 receptor, linked to Th2 pathogenicity and tissue inflammation (12, 13), CD38, responsible for cell adhesion and activation (67), peroxisome proliferator-activated receptor program gamma (PPARg) (5, 65), and CCR4, associated with inflammation and tissue homing (68), further refine this phenotype. A summary of phenotypic and functional markers associated with pathogenic Th2 cells across distinct contexts is summarized in Table 1 and Fig. 1.

**TABLE 1:** Phenotypic and functional markers associated with pathogenic Th2 cells across different contexts^*a*^^,^^*b*^

Marker	Expression in pathogenic Th2 cells	Associated disease context	Species	References
CCR4	+	Airway inflammation	Human/murine	(14, 68)
CCR8	+	AD	Murine	(11)
CD27	−−	CRS, EoE, EGID, food/environmental allergy	Human	(47–52)
CD38	+	Allergy	Human	(67)
CD45RA	−−	EoE	Human	(50)
CD45RB	−−	CRS, allergy	Human	(9, 49)
CD45RO	+	Asthma	Human	(43)
CD49d	+	CRS, food/ environmental allergy	Human	(9, 49, 50)
CD62L	−	Eosinophilic airway inflammation, allergy	Murine	(39, 41)
CD161	++	CRS, AD, EGID, EoE, asthma, food/ environmental allergy,	Human	(43, 48–50, 59, 61)
CRTH2	++	CRS, AD, EGID, EoE, asthma, food/ environmental allergy	Human/murine	(12, 43, 48–50, 55, 59–61, 69)
CXCR3	−	Eosinophilic airway inflammation, allergy	Murine/human	(39, 41, 68)
HPGDS	+	AD, food allergy	Human	(5, 65)
IL-4	++	AD, food/ environmental allergy	Murine/human	(11, 58, 60, 70)
IL-5	++	AD, CRS, EoE, asthma, eosinophilic airway inflammation, food/ environmental allergy	Murine/human	(11–13, 39, 60, 70, 71)
IL-13	++	AD, EoE, asthma, eosinophilic airway inflammation, food allergy	Murine/human	(11–13, 70)
IL-9R	+	Asthma	Human	(13)
IL-17RB	+	CRS, EoE, AD, food allergy	Human	(12, 53–55)
PPARg	+	Allergy	Human	(5, 65)
ST2	++	CRS, AD, food/ environmental allergy, asthma	Human/murine	(41, 43, 53–55, 72)
CD40L	++	Food/environmental allergy, asthma	Human/murine	(58)
GATA3	++	Master regulator of Th2 identity	Human/murine	(73)
CD127	++	Chronic airway inflammation	Murine	(74)
GPR15	+	Ulcerative colitis	Human	(75)

^*a*^ EoE, eosinophilic esophagitis; AD, atopic dermatitis; CRS, chronic rhinosinusitis; EGID, eosinophilic gastrointestinal disorders.

^*b*^ Expression: −−, negative; −, low; +, positive; ++high.

An interesting suggestion presented by Wambre et al. (9) is that at least three markers should be used to define these pathogenic Th2 cells: one associated with the differentiation stage, together with two additional markers from the broader pool described above, chosen according to the biological context and experimental platform. However, with the advent of high-dimensional technologies, particularly single-cell RNA sequencing, it has become clear that Th2 populations are far more heterogeneous than previously appreciated, requiring not only surface markers for annotation but also integrated gene and protein expression profiles to accurately define their molecular programs (56). Many commonly used markers, such as CRTH2, CD161, CCR4, ST2, and IL-17RB, are neither exclusive to pathogenic Th2 subsets nor stably expressed across tissues and disease states, and phenotypically similar populations can display markedly distinct functional properties depending on their transcriptional and epigenetic states (5, 17). Consequently, reliance on fixed immunophenotypic gates may obscure biologically meaningful heterogeneity or lead to misclassification of context-dependent Th2 populations (9, 10).

### Differentiation and maintenance

Beyond phenotypic definition, pathogenic Th2 cells are distinguished by the signals that drive their differentiation, stabilize their effector program, and sustain their persistence within inflamed tissues. Accumulating evidence indicates that these cells do not arise simply through canonical Th2 polarization but instead reflect a specialized differentiation trajectory shaped by chronic antigen exposure, tissue-derived cues, and survival-promoting signals that collectively bias Th2 immunity toward pathology (9, 41, 58, 64, 76).

Notably, the conversion of naïve CD4^+^ T cells into Th2 cells in lymph nodes is an important step in any adaptive type 2 immune response. Although it is well established that priming and differentiation of naïve CD4^+^ T cells toward the Th2 lineage requires allergen/antigen presentation by type 2 conventional dendritic cells (cDC2s), the instructive signals that drive this process remain incompletely defined (77). cDC2 subsets are known to produce cytokines and metabolites that promote differentiation of naïve CD4^+^ T cells into Th1 and Th17 effectors, as well as regulatory T cells (Tregs) (78); however, in the context of Th2 responses, the identity of equivalent polarizing factors, or even whether a dedicated Th2-skewing signal is required, remains unclear. Nonetheless, IRF4^+^ CD301b^+^ cDC2s have been shown, in human and mouse models, to play a critical role in Th2 priming (78, 79).

After differentiation, one of the earliest models to explain pathogenic Th2 cell plasticity highlighted the capacity of these cells to respond directly to epithelial-derived signals (9, 58, 64). In response to allergens, pathogens, and environmental chemicals, epithelial cells secrete a set of alarmins—including Thymic Stromal Lymphopoietin (TSLP), IL-33, and IL-25—that function as danger signals and initiate type-2 immune activation, in part, through dendritic-cell activation (80). TSLP canonically acts on dendritic cells to enhance costimulatory capacity and promote Th2 polarization (81, 82); however, epithelial alarmins alone are sufficient to skew Th2 differentiation toward a pathogenic phenotype, with TSLP-rich tissue environments preferentially driving the maturation of pathogenic effector Th2 cells over conventional Th2 (9). Moreover, the combined presence of IL-33 and IL-25 was associated with the generation of Th2 cells with enhanced effector function, characterized by robust production of IL-5 and IL-9 (83). Mechanistically, IL-33 stimulation triggers p38 MAP kinase signaling in memory Th2 cells, thereby licensing high-level IL-5 production and conferring pathogenic effector capacity, a pathway that has been validated *in vivo* during helminth infection (84).

Wambre et al. (64) formalized this framework by proposing that, in the context of chronic antigen exposure typical of allergic settings, these pathogenic Th2 cells acquire an innate-like program, reflecting their ability to integrate epithelial-derived alarmin signals independently of classical TCR engagement. Interestingly, this pathogenic program was shown to be uncoupled from canonical IL-4/IL-13 signaling (85), which is essential for initial Th2 lineage differentiation (2, 86). It was observed that blockade of IL-4Rα in allergic mice models aborted IgE production but did not eliminate or revert the pathogenic phenotype (85).

More recently, the application of high-resolution approaches such as single-cell RNA sequencing in allergic mouse models has revealed that, in addition to epithelial alarmins, members of the tumor necrosis factor receptor superfamily (TNFRSF) act as critical co-drivers of pathogenic Th2 differentiation (87). It was shown that co-stimulation through TSLP and TNFRSF members was sufficient to generate pathogenic Th2 cells *in vitro* that were transcriptionally and functionally highly similar to pathogenic Th2 cells isolated from inflamed tissues *in vivo* (87). At the molecular level, signaling through TSLP and TNFRSF converged to attenuate the activity of histone deacetylase 1 (HDAC1), resulting in increased chromatin accessibility at key Th2 cytokine loci, including *Il5* and *Il13* (87). This epigenetic remodeling facilitated stable, high-level expression of type-2 effector cytokines, thereby providing a mechanistic link between tissue-derived inflammatory cues, costimulatory signaling, and the fixation of a pathogenic Th2 effector program.

Together, these findings indicate that pathogenic Th2 cells are not only programmed by epithelial and costimulatory cues but are also functionally adapted for long-term residence within inflamed tissues, raising the question of which molecular mechanisms actively retain these cells at sites of chronic type-2 inflammation. In addition to intrinsic features such as the expression of chemokine receptors and adhesion molecules that support tissue homing and positioning, a non-canonical retention mechanism has been proposed (9, 11, 68). Specifically, the CD69–Myl9 axis was described not as a driver of pathogenic Th2 differentiation, but as a system that promotes their recruitment to and persistence within inflamed tissues. Although CD69 is not classically considered a defining marker of pathogenic Th2 cells, it is a well-established early activation marker of leukocytes and has been reported on subsets of activated Th2 cells and tissue-resident memory T cells (88). In this context, the interaction between CD69 and myosin light chain-9 (Myl9) was shown to be critical for facilitating T-cell trafficking into sites of inflammation (89). The proposed model suggests that, within inflamed tissues, activated platelets release Myl9, which polymerizes into net-like structures along the luminal surface of blood vessels (45). CD69-expressing T cells can recognize and bind to these Myl9 nets, thereby enabling their adhesion within the vasculature and subsequent transmigration into the tissue parenchyma (45). Through this mechanism, pathogenic Th2 cells would be preferentially recruited to and retained within inflamed tissue niches, ultimately contributing to the maintenance of chronic Th2 inflammation and disease persistence (45).

An additional, complementary mechanism proposed to support the maintenance of pathogenic Th2 cells involves IL-7 signaling within inducible bronchus-associated lymphoid tissue (iBALT) (41, 76, 90). iBALT is an organized tertiary lymphoid structure that forms adjacent to the bronchi in response to chronic pulmonary inflammation and is composed of distinct B-cell follicles with follicular dendritic cells, T-cell/dendritic cell zones, and specialized vasculature including high endothelial venules (91). Beyond serving as a site for local antigen presentation, iBALT provides a stromal niche that supports effector memory T cell survival. Within iBALT, lymphatic endothelial cells produce IL-7, which delivers potent anti-apoptotic signals that promote the persistence and longevity of Th2 cells in the tissue microenvironment (41, 76, 90). IL-7’s role in suppressing pro-apoptotic pathways helps sustain pathogenic Th2 populations independent of continued antigen recruitment from the circulation.

These observations raise an important conceptual question regarding the relationship between pathogenic Th2 cells and tissue-resident memory (Trm) cells. Multiple studies in allergic diseases have shown that pathogenic Th2 populations share several canonical features of Trm biology, including reduced recirculation capacity and long-term persistence within peripheral tissues (42), expression of CD69 (89), and dependence on local survival niches (41, 76, 90). However, whether pathogenic Th2 cells represent a specialized subset of Trm cells or instead acquire partial tissue-residency programs during chronic effector differentiation remains unresolved. Tissue residency may reinforce and stabilize pathogenic effector function by enabling sustained exposure to local inflammatory and survival signals, but tissue residency alone is unlikely to be sufficient to define pathogenicity. Pathogenicity and tissue residency are, therefore, best viewed as overlapping but non-equivalent programs, whose relationship likely depends on tissue context, chronicity of antigen exposure, and the local inflammatory microenvironment.

In summary, these recruitment and survival mechanisms establish a framework in which pathogenic Th2 cells are durably rooted within inflamed tissues, setting the stage for their sustained effector activity and the downstream functional consequences that drive chronic type-2 immunopathology.

### Pathogenic activity

Why, then, are these highly differentiated Th2 subsets termed pathogenic? Across allergic disease contexts, their sustained presence has been directly linked to core features of tissue pathology, including mucus hypersecretion (13, 14, 42), epithelial barrier dysfunction (13, 90), airway hyperresponsiveness (42), and progressive tissue remodeling and fibrosis (10, 43, 72). Multiple studies indicate that these pathological changes can be driven by the effector activity of pathogenic Th2 cells themselves, even in the absence of ongoing antigen exposure, leading to the proposal that the mere persistence of these cells is sufficient to initiate and maintain disease pathogenesis in type-2-mediated inflammatory disorders (9).

One of the clearest illustrations of this concept comes from single-cell transcriptomic profiling of airway epithelial cells from patients with asthma. It was demonstrated that type-2 inflammation drives not only classical goblet-cell hyperplasia but also the emergence of a previously unrecognized “mucous ciliated” epithelial cell state (13). In this study, ciliated epithelial cells exposed to a type-2 cytokine milieu acquired a hybrid transcriptional program characterized by retention of core ciliary genes alongside the induction of canonical goblet-cell-associated mucin and secretory genes, including MUC5AC, SPDEF, and AGR2 (13). This mucous ciliated phenotype, therefore, represents a form of mucus metaplasia within the ciliated epithelial compartment rather than a simple expansion of conventional goblet cells, providing a direct mechanistic link between sustained type-2 effector signaling and pathological epithelial remodeling. A second illustrative example of the pathogenic potential of these cells comes from their role in promoting tissue fibrosis in patients with eosinophilic chronic rhinosinusitis. In the context of eosinophilic inflammation, a specialized subset of pathogenic Th2 cells was shown to produce the epidermal growth factor family member amphiregulin (43). Rather than acting directly on stromal cells, Th2-derived amphiregulin functioned as a paracrine signal that reprogrammed eosinophils toward a pro-fibrotic phenotype (43). Specifically, amphiregulin induced eosinophils to upregulate osteopontin, a matricellular protein with well-established roles in fibroblast activation, extracellular matrix deposition, and tissue remodeling (92). Osteopontin production by eosinophils was both necessary and sufficient to drive fibrotic remodeling *in vivo*, thereby identifying a Th2–eosinophil–osteopontin axis as a critical effector pathway linking type-2 immunity to fibrosis.

Recently, beyond mechanistic studies, the pathogenic relevance of these highly differentiated Th2 cells has been reinforced by multiple clinical trials in which therapeutic strategies that reduce these populations are associated with meaningful clinical improvement (51, 65, 70, 71, 93–98). One demonstration comes from the Gauging Response in Allergic Rhinitis to Sublingual and Subcutaneous Immunotherapy (GRASS) trial, a randomized, double-blind, placebo-controlled study in adults with moderate-to-severe seasonal allergic rhinitis (98). Participants received either daily sublingual grass-pollen tablets or monthly subcutaneous grass-pollen injections for two years. Both treatment arms showed a significant reduction in allergen-reactive CRTH2^+^CD161^+^CD27⁻ memory Th2 cells after 1 year, and the magnitude of this reduction closely correlated with improvements in nasal allergen-challenge responses and clinical symptom scores (94). Importantly, after treatment cessation, both the frequency of these cells and the clinical benefits rebounded, indicating that persistence of pathogenic Th2 cells tracks directly with disease activity (94). A similar pattern was observed in house-dust-mite-driven allergic rhinitis, where 1 year of sublingual immunotherapy led to a selective reduction in allergen-reactive IL-5^+^IL-13^+^CD27⁻CD161^+^ CD4^+^ T cells, again correlating with clinical response (71).

Evidence for this principle extends to food allergy. A single intravenous dose of etokimab, an anti-IL-33 monoclonal antibody, increased peanut-protein tolerance and was associated with reduced frequencies of IL-4^+^IL-5^+^IL-9^+^IL-13^+^ST2^+^ CD4^+^ T cells (70). In a separate trial, combination therapy with omalizumab, a monoclonal antibody used to reduce circulating IgE antibodies (99), plus multi-allergen oral immunotherapy resulted in a marked contraction of pathogenic Th2 cells alongside induction of regulatory markers such as C1GALT1 and STAB1 (97). These findings suggest that the disruption of the IgE-dependent inflammatory milieu may indirectly limit the maintenance or persistence of pathogenic Th2 populations during chronic allergic inflammation. Finally, multiple other oral immunotherapy strategies for peanut allergy, including AR101 (Palforzia—defatted peanut*—Arachis hypogaea* flour) and PVX108 (pool of immunogenic peptides), increased tolerance thresholds and reduced reaction severity in parallel with sustained decreases in pathogenic Th2 cell frequencies (65, 95).

## PATHOGENIC Th2 CELLS IN HELMINTH INFECTIONS

### Th2-driven pathology in helminth infection

Helminth infections are among the most potent natural inducers of type 2 immunity in humans and experimental models. Canonical Th2 responses, defined by IL-4, IL-5, and IL-13 production, contribute to host defense by promoting eosinophil differentiation and recruitment, mucus production, smooth-muscle contractility, and antibody class switching, thereby supporting parasite expulsion and limiting tissue invasion (23, 100, 101). Accordingly, Th2 cells have traditionally been viewed as broadly protective in helminth infection, with emphasis placed on their roles in parasite control and in restraining excessive Th1/Th17-associated inflammation (23). In contrast to these effector functions, chronic helminth infection is characterized by robust induction of IL-10, produced by Th2 cells alongside regulatory T cells, which plays a central role in dampening inflammation, limiting immunopathology, facilitating worm expulsion, and promoting host–parasite tolerance (26, 29–32). IL-10-mediated regulation not only constrains anti-helminth immunity but also suppresses bystander responses to allergens, autoantigens, and unrelated pathogens, contributing to the systemic immunomodulatory effects that characterize chronic helminth infection (28).

Despite this protective framing of Th2 immunity during helminth infection, a substantial body of literature has emerged linking type 2 immune responses to helminth-associated pathology (21, 22, 100, 102–105). In schistosomiasis, parasite egg-induced granuloma formation in the liver represents one of the most well-characterized examples of helminth-driven immunopathology. In the liver, repeated granuloma formation around trapped eggs leads to progressive periportal fibrosis, hepatosplenomegaly, and obstruction of portal blood flow, resulting in portal hypertension and life-threatening complications such as variceal bleeding (106, 107). Experimental studies established that granuloma formation is predominantly driven by Th2 immunity, with IL-13 playing a central role (21, 22). Sustained IL-13 signaling directly promotes fibroblast activation and excessive collagen deposition, driving progressive fibrotic remodeling (21).

Beyond schistosomiasis, helminth-driven pathology is frequently observed in the lung, since the life cycles of many helminths undergo larval migration through the pulmonary vasculature and subsequently the parenchyma. Species such as *Ascaris* sp., hookworms (*Necator* and *Ancylostoma*), *Strongyloides* sp., and *Paragonimus* sp., all involve a lung phase that exposes pulmonary tissues to sustained type 2 immune activation. Experimental infection with *Nippostrongylus brasiliensis* has been shown to induce two temporally and mechanistically distinct forms of chronic pulmonary pathology, including impaired lung function, and destruction of lung parenchyma (103, 104). During the early phase of larval migration, lung inflammation is characterized by airway mucus production, eosinophil and lymphocyte infiltration, and Th2 cytokine production, all of which are dependent on IL-4Rα signaling in CD4^+^ T cells since mice with CD4^+^ T cell-specific IL-4Rα deficiency exhibit markedly reduced early lung inflammation despite normal parasite expulsion (104). However, the chronic pathology that emerges weeks later, including progressive alveolar destruction, impaired lung function, and emphysema-like changes, is driven by alternatively activated macrophages producing MMP-12 and is independent of IL-4R, IL-5R, and IL-13 signaling (103). Another illustrative example is *Ascaris* infection, which has been shown in animal models to induce airway hyperresponsiveness (AHR) and lung pathology resembling allergic asthma (108). Notably, AHR coincided with the peak of larval migration through the lung but persisted even after parasite clearance, indicating that pulmonary pathology can be sustained independently of ongoing infection (108). These effects were associated with robust type 2 cytokine responses in both bronchoalveolar lavage fluid and lung tissue, with particularly high levels of IL-5, reported to be approximately 10-fold greater than those observed in classical allergic airway inflammation (108).

Interestingly, helminth-induced pulmonary pathology is not restricted to species that directly traverse the lung parenchyma. Filarial infections such as *Wuchereria bancrofti* and *Brugia malayi*, whose microfilariae predominantly circulate within the pulmonary vasculature, can give rise to a severe immunopathological condition known as tropical pulmonary eosinophilia (TPE) (109). TPE is characterized by an exaggerated eosinophil-dominated inflammatory response in the lower respiratory tract, leading to chronic cough, wheeze, and pulmonary infiltrates (109, 110). This (TPE) syndrome is associated with heightened Th2 cell activation, with sustained production of IL-5 and elevated IgE levels promoting eosinophil recruitment, activation, and prolonged survival within the lung (109). Beyond pulmonary models, direct evidence linking CD4^+^ T cell effector function to helminth-associated tissue damage has emerged from experimental filarial infection studies, in which depletion of CD4^+^ T cells in a murine model of *Onchocerca volvulus* reduced ocular pathology, keratitis, and associated eosinophil recruitment (111).

The gut represents another major site of helminth-associated pathology, particularly for soil-transmitted helminths that complete their life cycle within the intestinal lumen or mucosa (20). Helminth infection has been shown to induce structural alterations in epithelial tight junctions and changes in the expression of junctional proteins such as E-cadherin, resulting in increased epithelial permeability (112, 113). These epithelial alterations are closely linked to type 2 immune signaling since they are abrogated in infected STAT6-deficient mice (112, 113).

These observations establish that sustained Th2 responses in helminth infection can drive tissue pathology through mechanisms strikingly analogous to those described in allergic disease, including eosinophil activation, epithelial remodeling, and fibroblast-driven extracellular matrix deposition. Yet, in contrast to allergy, these pathogenic outcomes have rarely been interpreted through the lens of Th2 cellular heterogeneity or attributed to specialized pathogenic Th2 subsets.

### Evidence for pathogenic Th2 cells in helminth infection

Given the extensive parallels between allergic and helminth-associated type 2 pathology, a critical unresolved question is whether pathogenic Th2 programs are generated during chronic helminth infection, or whether such programs are actively suppressed or simply remain under-characterized. Several studies in helminth-infected humans and experimental models have described Th2 populations with features highly reminiscent of pathogenic Th2 cells, including stable IL-5 expression, responsiveness to epithelial alarmins, expression of CRTH2 and CD161, and enrichment for tissue-homing and survival-associated markers (56, 114–119). However, these cells have almost uniformly been interpreted within a generic Th2 framework, without being explicitly conceptualized as a distinct pathogenic subset.

One illustrative example comes from murine schistosomiasis. Following intravenous challenge with *Schistosoma mansoni* egg antigen, CCR8^+^CD4^+^ T cells become enriched within pulmonary granulomas, and their accumulation correlates with increased eosinophil recruitment (117, 118). Critically, although these cells do not recapitulate the full cytokine profile of allergic pathogenic Th2 populations, producing IL-10 rather than IL-5 or IL-13 as their dominant output (117), their selective enrichment at a site of inflammation mirrors the pathology-associated tissue residency that defines pTh2 cells in allergic diseases, including eosinophil traffic to the lungs. A parallel pattern is observed in human helminth infection studies. Following controlled infection with 50 *Necator americanus* larvae, a phenotypically distinct memory CD4^+^ T-cell population expressing CD38 becomes detectable as early as 4 weeks post-infection and remains stable through at least 12 weeks (116). The functional maturation of this compartment is temporally delayed since robust cytokine production is not observed until approximately 8 weeks post-infection, and this emergence of effector activity coincides with the peak of gastrointestinal symptoms (116). As with the murine schistosomiasis model, this temporal coupling between the appearance of distinct Th2-like effector population and the onset of clinical pathology point to a shared principle: that specialized Th2 subsets, even when not phenotypically identical to those described in allergy, may mark or contribute to tissue-level disease consequences during helminth infection.

However, one of the first clear indications that helminth infection may be associated with the expansion of pathogenic Th2-like subsets emerged from high-dimensional mass cytometry profiling of CD4^+^ T cells in individuals infected with soil-transmitted helminths (*Ancylostoma duodenale*, *Necator americanus*, *Ascaris lumbricoides*, *Trichuris trichiura*, or *Strongyloides stercoralis*) (114). Compared with unexposed controls, infected individuals showed selective expansion of a CD27⁻CRTH2^+^CD161^+^ memory subset, a phenotype that closely resembles pathogenic Th2 populations described in allergic disease (114). This expansion was observed in a population with low prevalence of severe allergic pathology (120), arguing against allergy as the primary driver of this subset and instead implicating helminth exposure itself. A related pattern was observed in a hookworm vaccine context. Following *Na*-GST-1 vaccination in helminth-exposed individuals, antigen-responsive memory CD4^+^ T cells were enriched for CD161 expression (115). CD161 marks a highly differentiated effector state associated with eosinophil-driven inflammation and prolonged cell survival in allergic diseases, and its co-expression with CRTH2 and absence of CD27 defines the core pathogenic phenotype across multiple allergic contexts (6, 56, 121). Notably, this subset also co-expressed CTLA-4 and CD40L, molecules involved in the regulation and T-cell activation (122, 123) and which are not typically emphasized as defining features of classical pathogenic Th2 cells. Functional blockade of CTLA-4 enhanced cytokine production by the CD161^+^ memory compartment, suggesting that helminth-associated pathogenic Th2-like populations may retain intrinsic regulatory constraints (115), emphasizing the need to understand these populations within different microenvironments. These findings suggest that helminth-associated CD161^+^ Th2-like populations share phenotypic hallmarks of pathogenic Th2 cells but whether these cells contribute directly to helminth-associated pathology remains an open and important question that direct functional studies will need to address.

Additional support for helminth-associated expansion of pathogenic Th2-like programs comes from studies of human filarial infection. In this setting, infected individuals exhibited the expansion of a highly differentiated effector CD4^+^ T cell populations defined phenotypically as CD45RA⁻CCR7⁻CD127^+^CXCR3⁻CCR4^+^CRTH2^+^, comprising either CCR6^−^ or CCR6^+^ subsets enriched for markers previously associated with pathogenic Th2 cells (56). Beyond their phenotype, these subsets are functionally polarized, producing high levels of filarial antigen-specific IL-4, IL-5, and IL-13 (56). Notably, both Th2 cell subsets were further amplified in filaria-infected individuals with concurrent allergic disease. The authors demonstrated that pre-existing allergic sensitization reshaped the transcriptional and functional programming of CD4^+^ T cells triggered by filarial parasites toward a pathogenic Th2 immunophenotype, characterized by the upregulation of canonical pathogenic Th2-associated genes, including IL17RB, KLRB1, PTGDR2, PPARG, HPGDS, IL4, IL5, and IL13, alongside the downregulation of CD27 (56).

Together, these findings provide direct evidence that helminth infection can support the emergence of highly differentiated Th2 subsets that molecularly and functionally resemble pathogenic Th2 cells described in allergy. However, clear context-dependent differences exist among these populations, underscoring the need for careful evaluation of the local microenvironment, immunological landscape, and pathological outcomes when interpreting their function.

### Context-dependent functions of pathogenic Th2-like cells in helminth infections

Beyond the differences noted above between canonical allergic pathogenic markers and those observed in helminth settings, several studies describe Th2 subtypes that express hallmark pathogenic features yet exert protective or beneficial functions during infection (124–128). In murine helminth models, high IL-17RB expression on intestinal Th2 cells during *Trichinella spiralis* (125) or *Heligmosomoides polygyrus* (126, 127) infection is required for effective parasite-specific immunity, acting as a critical receptor for IL-25-driven worm expulsion. Similarly, memory ST2^+^IL-5^+^ Th2 cells limit the fecundity of *Nippostrongylus brasiliensis* (124), and T cell-intrinsic PPARγ signaling is necessary for differentiation into IL-5 and IL-13 producing effectors capable of mounting protective responses against *H. polygyrus* (128).

Human studies further highlight context-dependent divergence. During controlled *Necator americanus* infection, despite the increase in CD38^+^ cells, CD161^+^ effector memory CD4^+^ were diminished in infected individuals (116). This difference could be associated with the fact that controlled infection only exposes the individuals to a limited and transient infection, which does not correspond to what is true in endemic areas. Also, even when transcriptional programs strongly resemble allergic pathogenic Th2 signatures, important distinctions remain. In filaria-allergic individuals, effector memory T cells downregulate several canonical pathogenic markers, including CCR4, CCR8, IL5, IL9R, and KLRB1, while upregulating other components of the program (56). These findings reinforce that the expression of pathogenic Th2 markers does not uniformly predict pathological function, and that helminth-associated Th2 programs must be interpreted within their specific biological context.

Taken together, these studies highlight the important role of pathogenic Th2 cells in the biology of helminth infections. However, whether these cells primarily promote parasite expulsion or contribute to immunopathology remains incompletely understood. Moreover, the balance between protection and pathology may not be determined solely by intrinsic differences among Th2 cell subsets but also by Th2 cell-extrinsic mechanisms operating within local tissue immune networks. One possibility is that the magnitude and balance of type 2 cytokines, including IL-4, IL-5, and IL-13, produced collectively by Th2 cells, ILC2s, and eosinophils critically shape infection outcomes. In this model, coordinated type 2 responses promote parasite control and tissue repair, whereas excessive or dysregulated cytokine production drives immunopathology, chronic inflammation, and tissue dysfunction (129, 130).

## FUTURE DIRECTIONS

The role of pathogenic Th2 cells in helminth infection remains largely unexplored. Yet, given the substantial mechanistic overlap between allergic disease and helminth-driven type 2 pathology, these populations warrant focused investigation. Th2 responses in helminth infection are highly heterogeneous, shaped by differences in parasite species, tissue niche, and chronicity of exposure.

High-resolution technologies, including single-cell and other high-dimensional microscopy approaches, will, therefore, be essential to resolve this complexity and to determine whether pathogenic Th2 programs analogous to those described in allergy are generated, restrained, or repurposed in parasitic settings.

Helminth-induced immunity is profoundly influenced by local tissue microenvironments, yet most studies rely on dissociated cells that lose critical information about anatomical localization and cell–cell interactions. The emergence of spatial transcriptomics, localization, and related imaging-based single-cell approaches offers an unprecedented opportunity to address this gap. These technologies can preserve native tissue architecture while simultaneously defining the molecular identity of Th2 subsets, enabling visualization of their positioning relative to epithelial, stromal, and myeloid compartments that shape their function. Spatially resolved analyses will be particularly important in light of recent work describing “tissue microniches” that orchestrate type 2 immunity. A recent study demonstrated that protective and pathogenic type 2 responses are organized within discrete immunological hubs composed of defined combinations of immune and structural cells (131). Additionally, emerging evidence indicates that mucosal immunity is governed by tightly integrated epithelial–immune–neuronal circuits that regulate inflammation, repair, and barrier integrity, rather than by immune cells acting in isolation (132–135). Barrier epithelial cells actively sense environmental cues and induce type 2 responses through cytokine release, while neuronal signals further tune immune activation and tissue remodeling (132, 135). Incorporating this framework into spatial analyses of helminth infection could clarify how local circuit level interactions within microniches shape the differentiation, stability, and effector functions of pathogenic Th2-like populations.

Finally, future efforts should aim to build integrative atlases that combine single-cell transcriptomics, spatial mapping, and functional validation across diverse helminth species and tissues. Such resources would enable the field to move beyond a simplified view of “the Th2 response” toward a nuanced understanding of how distinct Th2 programs are deployed, maintained, or suppressed within defined anatomical and ecological contexts that define how Th2 cells mediate and/or contribute to protective versus pathological anti-helminth responses.
